# Advanced HIV disease and associated factors among young people aged 15—24 years at a tertiary hospital in Sierra Leone: a cross-sectional study

**DOI:** 10.1186/s12879-024-09524-5

**Published:** 2024-06-20

**Authors:** Mamadu Baldeh, Samuel Kizito, Sulaiman Lakoh, Daniel Sesay, Samuel Adeyemi Williams, Umu Barrie, Frida Dennis, Dimbintsoa Rakotomalala Robinson, Franck Lamontagne, Franck Amahowe, Patrick Turay, Ozge Sensoy Bahar, Elvin Geng, Fred M. Ssewamala

**Affiliations:** 1 College of Medicine and Allied Health Sciences, University of Sierra Leone, Freetown, Sierra Leone; 2 Medical Research Council Unit The Gambia at London School of Hygiene and Tropical Medicine, Banjul, The Gambia; 3 Washington University in St. Louis, St. Louis, MO USA; 4 University of Sierra Leone Teaching Hospital Complex, Freetown, Sierra Leone; 5 ISD – Innovation et Solidarité pour le Développement, ISD – Innovation et Solidarité pour le Développement, Paris, France; 6 Solthis - Solidarité Thérapeutique Et Initiatives Pour La Santé, Freetown, Sierra Leone; 7 University of Makeni, Makeni, Sierra Leone

**Keywords:** HIV, Advanced HIV Disease, Sierra Leone, Youths, Antiretroviral therapy, CD4 + cell count

## Abstract

**Background:**

Advanced HIV disease (AHD) in young people living with HIV (PLHIV) is an increasingly pressing public health issue in sub-Saharan Africa. Despite global progress in early HIV testing and reducing HIV-related deaths, many youths experience increased rates of HIV disease progression in sub-Saharan Africa. This study describes the burden, clinical manifestations, and factors for disease progression among young PLHIV aged 15 – 24 years seeking medical services at a major public hospital in Sierra Leone.

**Methods:**

We performed a cross-sectional analysis of routinely collected data for PLHIV patients aged 15 to 24 seen at Connaught Hospital in Sierra Leone between September 2022 and March 2023. We estimated the proportion of AHD in young PLHIV and performed logistic regression modelling to explore predictors of AHD. The statistical significance level was set at 0.05 for all statistical tests.

**Results:**

Of the 581 PLHIV that were reported, 238 (40.9%) were between the ages of 15 and 24 years, with a median age of 22 (20—24), and 151 (63.5%) were females. On review, 178 (74.8%) has initiated antiretroviral therapy regimen (ART); 117 (65.7%) were actively on ART for ≤ 6 months, while 114 (64%) had interruptions with their ART treatment. The overall prevalence of AHD was 41.6% (99/238); 46.7% (35/68) of young PLHIV at the HIV clinic, and 39.3% (64/163) of admission. Sex—Female (OR, 0.51; 95% CI, 0.28–0.94; *p* = 0.030), and Tertiary Education level (OR, 0.27; 95% CI, 0.10 – 0.78; *p* = 0.015) have significantly lower odds of AHD in the entire study population. While for inpatients, Age (young Adults) of PLHIV (OR, 1.23; 95% CI, 1.00–1.52; *p* = 0.047) had 1.23 times the odds of AHD compared to adolescents, and being female (OR, 0.27; 95% CI, 0.08–0.84; *p* = 0.024), Overweight—Body mass index (OR, 0.10; 95% CI, 0.01–0.77; *p* = 0.028), Tertiary Education level (OR, 0.08; 95% CI, 0.01–0.52; *p* = 0.008) have significantly lower odds of AHD. Common conditions reported for the AHD group in the medical wards are tuberculosis (13.58%), hepatitis B (6.13%), Kaposi sarcoma (3.07%), and oesophagal candidiasis (2.45%).

**Conclusion:**

We reported a high prevalence of advanced HIV among young patients in a tertiary Hospital in Sierra Leone. One in two young PLHIV aged 15 to 24 years reported AHD, emphasizing the need to strengthen public health measures that address access to and retention of HIV services.

## Background

Human immunodeficiency virus (HIV) remains a major global health challenge, affecting millions of people worldwide. In 2022, an estimated 39 million people living with HIV (PLHIV) globally, with sub-Saharan Africa (SSA) carrying over 75% of the global disease burden [1, 2]. Despite the significant progress in scaling up HIV prevention, increased access to antiretroviral therapy (ART), and improved life expectancy of PLHIV, a systematic review of about 2,500 records showed an AHD prevalence of 43.5% among ART-naïve and 58.6% among ART-experienced [3]. Young people, also referred to as youths by the United Nations, aged 15 to 24 years account for 40% of new HIV infections; 2,400 get infected daily, and 5 million live with HIV [4].

In SSA, complex, multi-layered social dynamics and programmatic factors have been linked to the increased burden of AHD [5]. Despite late-stage diagnosis, issues related to linkage to care, treatment interruptions, and social challenges such as stigma and discrimination contribute to the disease progression [6–8] and the occurrence of opportunistic infections [8]. Although significant progress in the past decade has resulted in a 46% decrease in new HIV infections among youths aged 15–24 years, nearly 3 million young people with HIV present late to healthcare facilities for care. AHD, defined by the World Health Organization (WHO) as PLHIV having a CD4 < 200 cells/mm3 or stage 3 or 4 for adults, adolescents, and older children, is associated with a high risk of death and increased healthcare expenditure. Indeed, young people with AHD present a greater risk of mortality from causes related to HIV [6, 7, 9].

Sierra Leone, a Western African nation with a population of 8·5 million people, has a mixed HIV prevalence of 1.7%: higher in urban areas (2.3%) than in rural areas (1.2%) [10, 11]. National HIV programme is integrated into the healthcare system, primarily through provider-initiated testing at health facilities and targeted testing among vulnerable populations, families and key populations. PLHIV in Sierra Leone have access to free HIV care and support services, which are largely led by nurses and community health workers at drop-in and adolescent and youth centres.

Despite adopting the WHO recommendations for HIV testing and treatment, Sierra Leone still faces significant hurdles in achieving the 95–95-95 goal set by the Joint United National Programme on HIV/AIDS (UNAIDS); with only 76% of PLHIV knowing their HIV status and receiving antiretroviral therapy nationally. There continues to be a significant burden of undiagnosed HIV infection and advanced disease [12]. The advent of the 10-year civil war, the 2014–2016 Ebola epidemic, and other public health system challenges have made young people more vulnerable and, thus, have prompted the increased call to action to address the growing HIV epidemic nationally [13, 14].

Much of Sierra Leone’s population is relatively young, with an individual median age of 19.9 years; roughly one-fourth of the entire population falling within the age bracket of 15 to 24 years [15–17]. The impact of AHD on the well-being and socioeconomic status of young PLHIV in Sierra Leone is yet to be fully understood. Despite these deep-rooted public health challenges, a PubMed search in June 2023 found no data on the burden of AHD among young people aged 15–24 years living with HIV in Sierra Leone. Studying the level of AHD burden and identifying contributing factors in young people in Sierra Leone will aid global efforts to combat the HIV epidemic by developing well-informed policies and improving clinical practices. In this observational study, we explored the burden of AHD and associated risk factors among young PLHIV aged 15–24 years at a national referral hospital in Sierra Leone.

## Methods

### Study design and study setting

We employed a cross-sectional study design to analyze routinely collected data for PLHIV aged 15 to 24 years seen at Connaught Hospital, a national referral health centre in Freetown, Sierra Leone. The facility is part of the University of Sierra Leone Teaching Hospitals Complex, the main teaching affiliate of the College of Medicine and Allied Health Sciences. It offers various medical services to an extensive catchment population nationally. It also serves as the primary and largest HIV clinic in Sierra Leone. The HIV clinic database is a routine observational database of PLHIV who seek and receive outpatient and or inpatient care. This permits PLHIV monitoring, disease progression and outcome to ensure continuity of care and adherence to clinical management.

### Study population and sampling methods

Between September 2022 and March 2023, all identified PLHIV aged 15–24 years who reported to Connaught Hospital were included in the study. The following inclusion criteria were used: 1) aged 15–24 years; 2) diagnosed with HIV and status confirmed; and 3) sought HIV service at the facility. The HIV clinic at Connaught provides routine HIV services, including testing, follow-up and medication refills for PLHIV aged ≥ 15 years. PLHIV ≥ 18 years old who reported to the outpatient department or HIV clinic with major complaints for further management were admitted to the medical wards based on clinical findings.

### Measures and definitions

AHD was defined as any YLHIV with (i) CD4 + count below 200 cells/mm^3^ or (ii) WHO clinical stage 3 or 4. We determined HIV status using a third-generation rapid diagnostic test (Standard Diagnostics Bioline HIV -1/2 3.0) and CD4 cell count using the Alere Pima Analyzer. Kaposi sarcoma was diagnosed histologically through the Kaposi sarcoma program in Sierra Leone. We considered active tuberculosis (TB) when either of the criteria was met: Bacteriologic confirmation, Microscopy or GeneXpert; or clinically diagnosed by the consultant; or on anti-TB medication at the time of visit or admission. We used clinical features to confirm opportunistic infections such as oesophagal candidiasis, cryptococcal disease, mucocutaneous lesions and other coinfections (Table 1).

**Table 1:** Definitions of clinical diagnoses amongst PLHIV at Connaught Hospital

Advanced HIV Disease	I. CD4 + below 200 cells/uL II. World Health organization clinical stage classification: a. Stage 3 (moderately symptomatic stage): manifest weight loss > 10% of total body weight, pulmonary tuberculosis, mucocutaneous lesions, including recurrent oral candidiasis, severe systemic bacterial infections and prolonged unexplained diarrhoea b. Stage 4 (severely symptomatic stage): a manifestation of wasting syndrome, Pneumocystis pneumonia (PCP), recurrent severe or radiological bacterial pneumonia, extrapulmonary tuberculosis, chronic or oro-labial herpes simplex infection, oesophagal candidiasis, and Kaposi’s sarcoma
Tuberculosis	Active disease considered when either of criteria was met: I. Bacteriologic confirmation, Microscopy or GeneXpert II. Clinically diagnosed by the consultant III. On anti-TB medication at the time of visit or admission
Cryptococcal disease	Positive cryptococcal antigen test with meningeal or non-meningeal disease symptomatology
Candidiasis	Evidence of oral or oesophagal form diagnosed by the clinical team
Toxoplasmosis	Diagnosed by the clinical team
Kaposi sarcoma	Considered based on clinical team and histopathologic diagnosis
Weigh Loss / Malnutrition	Confirmed by anthropometric measurement (e.g., arm circumference, body mass index ≤ 18.5 kg/m^2^), or clinical signs of wasting syndrome
Anaemia	Haemoglobin concentration < 12.9 g/dl in males or < 11.9 g/dl in females
Co-infection	Diagnosis of co-infection with Mycobacterium tuberculosis, Cryptococcosis, hepatitis B virus, hepatitis C virus, and Plasmodium falciparum

ART status was classified as “Active,” “Interrupted,” or “Naïve” according to the 2018 national Differentiated care model. ART Naïve PLHIV (newly diagnosed or previously known HIV) had never received ART, while “Interrupted ART” PLHIV had experienced an interruption of their ART regimen for at least three months before being evaluated at the current hospital. PLHIV were classified as “Active on ART” if they had been on ART for at least three months prior to being seen at the hospital for evaluation.

### Study procedure

We extracted, cleaned and entered de-identified patient health records into password-protected Microsoft Excel databases. The clinical outcome was “AHD” or “No AHD” status based on WHO clinical staging and CD4 + count at the HIV clinic during routine follow-up visits or on admission to the medical wards at Connaught Hospital. We retrieved data on independent social-demographic variables, including age (15–19 years, and 20–24 years), sex, educational level, employment status (Student, Full-time and part-time), and recreational substance use. Clinical data retrieved includes diagnostic status (Newly diagnosed and Known case), ART Experience (Yes or No), Duration on ART (< 3 Months, 3–6 Months, > 6 Months, Unknown), ART interruption (Yes, No), CD4 + count, any reported symptoms, and opportunistic infections.

### Data management and analysis

Data was inputted into a Microsoft Excel spreadsheet. All statistical analyses were performed using Stata version 17.0. Descriptive statistics were computed to summarize the data. Continuous variables were summarized using mean (± standard deviation) or median (interquartile range) according to their distribution, while categorical variables were presented as frequencies and proportions. We stratified the sample into two categories: those with AHD and those without AHD. The two groups were compared using independent samples t-tests for continuous variables and chi-squared tests for categorical variables.

To identify the potential correlates of AHD, we fitted two separate logistic regression models. The first model included the entire sample, encompassing both inpatients and outpatients. In contrast, the second model was fitted exclusively for the sub-sample of inpatients. In the logistic regression models, AHD served as the dependent variable, which was dichotomized, with the presence of AHD coded as 1 and absence coded as 0.

The logistic regression assumptions were thoroughly checked, including linearity in the logit for continuous independent variables using the Box-Tidwell procedure, where each continuous variable is paired with its natural log term and the absence of multicollinearity. The goodness-of-fit of our logistic regression models was assessed using the Hosmer–Lemeshow test. In this test, a non-significant result indicates a good fit of the model, as it suggests no difference between the observed and predicted values of the dependent variable in our model. Given the considerable correlation between substance use and smoking, we included an interaction term for these two variables in our model. This enabled us to investigate whether the effect of substance use on AHD differed depending on whether the participant was a smoker. The interaction term was created by multiplying the substance use and smoking variables and then included in our logistic regression models. The statistical significance level was set at an alpha of 0.05 for all tests. We reported the odds ratios and the 95% confidence intervals.

### Ethics approval and consenting

Ethics approval was obtained from the Sierra Leone Ethics and Scientific Review Committee under the Reference SLESRC No. 032/09/2022. We obtained a waiver for informed consent from the Ethics committee and HIV Clinic, Connaught Hospital since we sought to use solely pre-existing, routinely collected, de-identified program data without requiring any interaction with participants.

## Results

### Baseline characteristics

Of the 581 PLHIV seen at Connaught Hospital between September 2022 and March 2023, 238 (40.9%) PLHIV were between the ages of 15 years and 24 years, with a median age of 22 years (IQR 20 -24 years), and of whom 151 (63.5%) were females. A total of 178 (74.8%) PLHIV aged 15–24 years had initiated ART before being seen at Connaught Hospital, of whom 117 (65.7%) were active on their treatment for at least 6 months, while 114 (64.0%) had documented interrupted ART treatment regimen. CD4 + cell count was documented for 188 (78.9%) of whom 64 (26.9%) had CD4 + cell count ≤ 200 cells/mm^3^. The median CD4 + cell count was 299 cells/mm^3^ (IQR 143–492 cells/mm^3^). Table 2 summarises the characteristics of all PLHIV aged 15–24 years seen at Connaught Hospital.

**Table 2:** Baseline characteristics for PLHIV seen at Connaught Hospital (September 2022 – March 2023)

	**15 – 19 years** ***N* = 43**	**20—24 years** ***N* = 195**	**Overall** ***N* = 238**	***P* value**
Median Age, years, (IQR)	18 (17—19)	23 (22- 24)	22 (20—24)	< 0.001*
Sex of the participant				
Male, *n* (%)	13 (30.2)	74 (37.9)	87 (36.6)	0.342
Female, *n* (%)	30 (69.8)	121 (62.1)	151 (63.4)	
Education level				
No education or primary, *n* (%)	10 (23.3)	40 (20.5)	50 (21)	0.016*
Secondary education, *n* (%)	32 (74.4)	111 (56.9)	143 (60.1)	
Tertiary level, *n* (%)	1 (2.3)	44 (22.6)	45 (18.9)	
Marital status				
Single, *n* (%)	43 (100)	181 (92.8)	224 (94.1)	0.070
Married, *n* (%)	0 (0)	14 (7.2)	168 (5.9)	
Employment status				
Student, *n* (%)	23 (53.5)	70 (35.9)	93 (39.1)	0.308
Fully employed, *n* (%)	0 (0)	7 (3.6)	7 (2.9)	
Part-time employment, *n* (%)	14 (32.4)	89 (45.6)	103 (43.3)	
Unemployed^a^, *n* (%)	6 (13.9)	29 (14.9)	35 (14.7)	
Recreation substance use				
Alcohol use, *n* (%)	8 (18.6)	45 (23.1)	53 (22.3)	0.523
Smoking, *n* (%)	7 (16.3)	39 (20)	46 (19.3)	0.673
illicit drug use, *n* (%)	5 (11.6)	30 (15.4)	35 (14.7)	0.529
Diagnostic stage				
Newly Diagnosed, *n* (%)	21 (48.8)	95 (48.7)	116 (48.7)	0.989
Known, *n* (%)	22 (51.2)	100 (51.3)	122 (51.3)	
ART Experience				
Yes, *n* (%)	32 (74.4)	146 (74.9)	178 (74.8)	0.951
No (Naïve), *n* (%)	11 (25.6)	49 (25.1)	60 (25.2)	
Duration on ART				
< 3 Months	5 (15.6)	23 (15.8)	28 (15.7)	0.308
3–6 Months	2 (6.3)	24 (16.4)	26 (14.6)	
> 6 Months	24 (75.0)	93 (63.7)	117 (65.7)	
Unknown	1 (3.1)	6 (4.1)	7 (4)	
Median weeks of ART interruption, IQR	6 (4 -8)	5 (4–7)	5 (4 – 7)	0.568
ART Interruption				
≤ 3 Months (Naïve), *n* (%)	4 (20.0)	23 (23.5)	27 (22.9)	0.433
4–6 Months, *n* (%)	7 (35.0)	46 (46.9)	53 (44.9)	
> 6 months, *n* (%)	8 (40.0)	26 (26.5)	34 (28.8)	
Not documented, *n* (%)	1 (5.0)	3 (3.1)	4 (3.4)	
Median CD4 + cell count (mm^3^, IQR)	276 (123–528)	299 (146—486)	299 (143.75–492)	0.023*
CD4 + Cell Count				
≤ 200cell/mm^3^, *n* (%)	10 (23.3)	54 (27.7)	64 (26.9)	0.460
> 200 cell/mm^3^, *n* (%)	21 (48.8)	103 (52.8)	124 (52.1)	
Not documented, *n* (%)	12 (27.9)	38 (19.5)	50 (21.0)	
WHO HIV clinical stage				
Stage 1, *n* (%)	16 (37.2)	65 (33.3)	81 (34.0)	0.762
Stage 2, *n* (%)	13 (30.2)	61 (31.3)	74 (31.1)	
Stage 3, *n* (%)	9 (21)	53 (27.2)	62 (26.1)	
Stage 4, *n* (%)	5 (11.6)	16 (8.2)	21 (8.8)	

^a^The unemployed included the unable to work, and those who do not fall in these two categories but are not working; the drugs included kush [7], Marijuana [9], and shisha [10]

A total of 75 (31.5%) PLHIV aged 15–24 years were seen at the HIV clinic as outpatients, of whom 43 (57.3%) were females, 163 (86.5%) were admitted to the medical wards as inpatients, of whom 108 (66.3%) were females. A total of 93 (39.1) were students, 34 (20.9%) consumed alcohol, while 35 (14.7%) of all PLHIV aged 15–24 years were involved in some form of illicit drug use (Table 3).

**Table 3:** Baseline characteristics for PLHIV aged 15–24 years seen at Connaught Hospital (September 2022 – March 2023)

	**HIV Clinic** ***N* = 75**	**Medical Ward** ***N* = 163**	**Overall** ***N* = 238**	***P* value**
Median Age, years, (IQR)	22 (20—23)	22 (20—24)	22 (20–24)	0.310
Sex of the participant				
Male, *n* (%)	32 (36.8)	55 (63.2)	87	0.184
Female, *n* (%)	43 (28.5)	108 (71.5)	151	
Education level				
No education or primary, *n* (%)	16 (32)	34 (68)	50	0.937
Secondary education, *n* (%)	45 (31.5)	98 (68.5)	143	
Tertiary level, *n* (%)	14 (31.1)	31 (68.9)	45	
Marital status				
Single, *n* (%)	72 (32.1)	152 (67.9))	224	0.402
Married, *n* (%)	3 (21.4)	11 (78.6)	14	
Employment status				
Student, *n* (%)	29 (31.2)	64 (68.8)	93	0.003*
Fully employed, *n* (%)	1 (14.3)	6 (85.7)	7	
Part-time employment, *n* (%)	31 (30.1)	72 (69.9)	103	
Unemployed^a^, *n* (%)	14 (40)	21 (60)	35	
Recreation substance use				
Alcohol use, Yes (*n*), (%)	19 (35.8)	34 (64.2)	53	0.441
Smoking, Yes (*n*), (%)	13 (28.3)	33 (71.7)	46	0.534
illicit drug use, Yes (*n*), (%)	6 (17.1)	29 (82.9)	35	0.048*
Diagnostic stage				
Newly Diagnosed, *n* (%)	60 (51.7)	56 (48.3)	116	0.001*
Known, *n* (%)	15 (12.3)	107 (87.7)	122	
ART Experience				
Yes, *n* (%)	15 (8.4)	163 (91.6)	178	< 0.001*
No, *n* (%)	60 (100)	0 (0)	60	
ART Interruption, Yes, *n* (%)	7 (5.9)	111 (94.1)	118	< 0.001*
Median weeks of Treatment interruption, IQR	6 (6)	5 (4—7)	5 (4—7)	< 0.001*
Median CD4 + cell count (mm^3^, IQR)	272.5 (169.8– 469.8)	303 (138.0–525.8)	299 (143.8–492)	0.9649
WHO HIV clinical stage				
Stage 1, *n* (%)	15 (18.5)	66 (81.5)	81	< 0.001*
Stage 2, *n* (%)	27 (36.5)	47 (73.5)	74	
Stage 3, *n* (%)	19 (30.6)	43 (69.4)	62	
Stage 4, *n* (%)	14 (66.7)	7 (33.3)	21	
Advanced HIV Disease				
Yes	35 (35.4)	64 (64.6)	99 (41.6)	0.045*
No	40 (28.7)	113 (81.3))	139 (58.4)	

^a^The unemployed included those who do not fall in these two categories but are not working; the drugs included kush [7], Marijuana [9], and shisha [10]

### AHD prevalence

A total of 99 (42.9%) of PLHIV aged 15 years to 24 years who reported to Connaught Hospital had AHD; 35 (35.4.7%) of the total AHD cases (99) were seen at the HIV clinic, while 64 (64.6% all AHD cases (99) admitted to the medical wards. A total of 83 (34.9%) of PLHIV aged 15–24 years were classified as either WHO clinical stage 3 or stage 4 HIV disease (Table 3).

Table 4 summarises the characteristics based on the disease stage of young PLHIV aged 15 to 24 years admitted at Connaught Hospital. Of the 163 PLHIV admitted to the medical wards, about 20% had received only primary or no education, while the majority (60%) achieved secondary school education. Almost half 15 (44.1%) of total PLHIV who reported alcohol intake had AHD. A total of 45 (27.6%) were active ART, of whom 8 (17.8%) had AHD, while 111 (68%) had experienced interrupted ART treatment, of whom 37 (33.3%) had AHD).

**Table 4:** Sociodemographic characteristics of 163 YLHIV admitted at Connaught Hospital

	**No AHD**	**AHD**	**Overall**	***P* value**
**Median Age (IQR)**	23 (20—24)	23 (21—24)	22 (20—24)	0.163
**Sex of the participant**				
Male, *n* (%)	30 (54.5)	25 (45.5)	55	0.248
Female, *n* (%)	69 (63.9)	39 (36.1)	108	
**Education level**				
No education or primary, *n* (%)	18 (56.2)	14 (43.8)	32	0.011*
Secondary education, *n* (%)	53 (54.1)	45 (45.9)	98	
Tertiary level, *n* (%)	26 (83.9)	5 (16.1)	31	
**Marital status**				
Single, *n* (%)	93 (61.2)	59 (38.8)	152	0.663
Married, *n* (%)	6 (54.5)	5 (45.5)	11	
**Employment status**				
Fully employed, *n* (%)	2 (33.3)	4 (66.7)	6	0.018*
Part-time employment, *n* (%)	37 (51.4)	35 (48.6)	72	
Not employed, *n* (%)	60 (70.6)	25 (29.4)	85	
**Recreation substance use**				
Alcohol use, *n* (%)	19 (55.9)	15 (44.1)	34	0.056
Smoking, *n* (%)	19 (57.6)	14 (42.4)	33	0.206
illicit drug use, *n* (%)	17 (58.6)	12 (1.44)	29	0.499
**Median number of sex partners (IQR)**	2 (1 – 2)	1 (1—3)	1 (1 – 2)	0.416
**Herbal medicine use**				
History of herbal medicine use, *n* (%)	67 (64.4)	37 (35.6)	104	0.201
No herbal medicine use, *n* (%)	32 (54.2)	27 (45.8)	59	
**Median weeks of illness (IQR)**	3 (2 – 4)	4 (2 – 5)	3.5 (2 – 4)	0.185
**ART experienced, *n* (%)**	111 (71.2)	45 (28.8)	156	0.017*
**ART status at admission**				
Active on ART	37 (82.2)	8 (17.8)	45	0.009*
ART Naïve	2 (28.6)	5 (71.4)	7	
ART Interruption	74 (66.7)	37 (33.3)	111	

The unemployed included student, and those who do not fall in these two categories but are not working; the drugs included kush [7], Marijuana [9], and shisha [10]

### Clinical presentations and co-morbidity among inpatients

Table 5 shows the clinical presentation of PLHIV aged 15–24 years at the time of admission to the medical wards Connaught Hospital. The majority reported or presented with more than one symptom: Weight loss (44.2%), fever (30.7%), cough (18.4%), headache (17.8%), and diarrhoea (8.0%) were the most common symptoms. Opportunistic infections included pulmonary TB (13.6%), Kaposi sarcoma (3.1%), and oesophagal candidiasis (2.5%). Other comorbid conditions included chronic hepatitis B (6.1%).

**Table 5:** Clinical characteristics of 163 YLHIV admitted at Connaught Hospital

Participant characteristics	Frequency (%)
**Common presenting symptoms**	
Weight loss	72 (44.2)
Fever	50 (30.7)
Cough	30 (18.4)
Headache	29 (17.8)
Diarrhea	13 (7.9)
Others^a^	71 (43.6)
**Body mass index**	
Underweight	44 (27.2)
Normal	98 (60.5)
Overweight	12 (7.4)
Obesity	8 (4.9)
**Admission diagnosis**	
Tuberculosis	22 (13.5)
Hepatitis B	10 (6.1)
Kaposi Sarcoma	5 (3.1)
Esophageal candidiasis	4 (2.5)
Others^b^	15 (9.2)
**CD4 count**	
CD4 + below 200	41 (34.2)
CD4 + more than 200	79 (65.8)
**WHO HIV clinical stage**	
Stage 1	66 (40.5)
Stage 2	47 (28.8)
Stage 3	43 (26.4)
Stage 4	7 (4.3)
**Advanced HIV disease**	
No advanced HIV	99 (60.7)
Diagnosed with advanced HIV	64 (39.3)

^a^Other symptoms include abdominal pain [16], genital discharge [14], difficult breathing [8], night sweats [7], genital ulcer [4], vomiting [3]

^b^Other admission diagnoses include gastric ulcers, herpes labialis, molluscum contagiosum, PPE, post-herpetic neuralgia, urticaria, vaginal ulcer syndrome

### Factors associated with advanced HIV disease

**Fig. 1:**
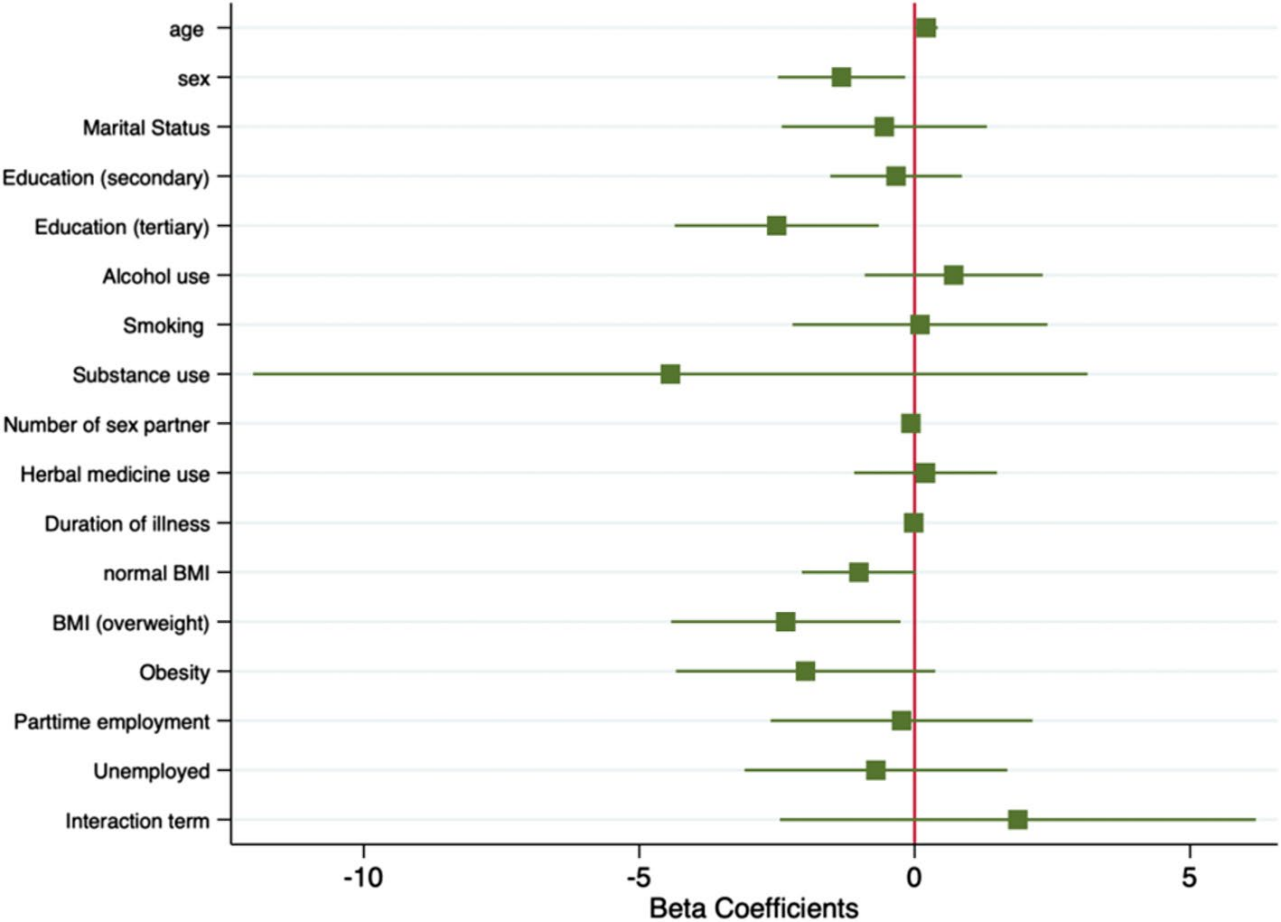
Forest plot for the logistic regression model assessing the association between various predictors and AHD among inpatients

A multivariate analysis showed that Sex—Female (OR, 0.51; 95% CI, 0.28–0.94; *p* = 0.030), and Tertiary Education level (OR, 0.27; 95% CI, 0.10 – 0.78; *p* = 0.015) have significantly lower odds (protective factors) of AHD in the entire study population. While for inpatients, Age (young adult aged 20–24 years) of PLHIV (OR, 1.23; 95% CI, 1.00–1.52; *p* = 0.047) and Sex—female (OR, 0.27; 95% CI, 0.08–0.84; *p* = 0.024), Overweight—Body mass index (OR, 0.10; 95% CI, 0.01–0.77; *p* = 0.028), Tertiary Education level (OR, 0.08; 95% CI, 0.01–0.52; *p* = 0.008) have significantly lower odds of AHD. (Table 6). Factors that predicted AHD included adolescents (age 15–19 years), male sex, lower educational level, and lower body mass index among inpatients (Fig. 1). WHO clinical stage, CD4 + count and treatment interruption were excluded from the multivariate analysis due to AHD definition criteria and high multicollinearity. The number of sexual partners, herbal medication and body mass index for PLHIV seen at HIV clinic as outpatient was not documented.

**Table 6:** Factors associated with AHD among YLHIV admitted to Connaught Hospital

	Entire sample (*n* = 238)			Inpatients (*n* = 163)		
Characteristics	Odds Ratio	95% CI	*P* value	Odds Ratio	95% CI	*P* value
Age	1.03	0.91 – 1.17	0.611	1.23	1.00 – 1.52	0.047*
Sex of the participant						
Male	1			1		
Female	0.51	0.28 – 0.94	0.030*	0.27	0.08 – 0.84	0.024*
Marital status						
Single	1			1		
Married	1.24	0.37 – 4.16	0.730	0.58	0.09 – 3.71	0.562
Education level						
No education or primary	1			1		
Secondary education	0.85	0.41 – 1.76	0.667	0.71	0.22 – 2.36	0.580
Tertiary level	0.27	0.10 – 0.78	0.015 *	0.08	0.01 – 0.52	0.008 *
Recreation substance use						
Alcohol use	1.31	0.56 – 3.09	0.533	2.03	0.40 – 10.23	0.390
Smoking	2.56	0.62 – 11.6	0.195	1.10	0.11 – 11.14	0.935
illicit drug use	0.60	0.01 –117.0	0.851	0.01	< 0.01 –23.07	0.251
Body mass index						
Underweight				1		
Normal				0.36	0.13 – 1.02	0.056*
Overweight				0.10	0.01 – 0.77	0.028 *
Obesity				0.14	0.01 – 1.45	0.099
Employment status						
Fully employed	1			1		
Part time employment	0.36	0.06 – 2.28	0.277	0.78	0.07 – 8.50	0.845
Not employed	0.30	0.05 – 1.95	0.205	0.49	0.05 – 5.38	0.563
Substance*smoking interaction	0.66	0.04 –12.36	0.783	6.51	0.09 – 4.89.34	0.395
	*Hosmer Lemeshow Goodness of fit: χ*^*2*^* (100)* = *122.81, p* = *0.061*			*Hosmer Lemeshow Goodness of fit: χ*^*2*^* (96)* = *109.35, p* = *0.166*		

^*^Denotes associations that are statistically significant

## Discussion

This is the first study in Sierra Leone and the sub-region to assess the burden and associated risk factors of AHD in young PLHIV aged 15 years to 24 years. In this young population with a median age 22 years (IQR 20 – 24 years), AHD prevalence was 42.9% (36.6%—49.4%) among 238 between September 2022 and March 2023 at the national referral hospital in Sierra Leone. Age (adolescent, 15–19 years), Female sex, lower educational level, low BMI and ART Interruption were independent predictors of AHD.

The high prevalence of AHD observed in young PLHIV aged 15 years to 24 years in our study is of significant concern. Despite Sierra Leone’s low HIV incidence of 1.7%, the prevalence of AHD (41.6%) in this study among PLHIV aged 15–24 years is higher than that reported in several other sub-Saharan African nations [18, 19]. A population-based cross-sectional study in South Africa, Kenya and Malawi reported a lower adult AHD prevalence of 9.7% [19], while another cross-sectional study in Kenya reported an AHD prevalence of 33% [20]. Our study setting, as a tertiary-level health facility, raises concerns about patient-related or health system-related issues that lead to poor healthcare-seeking behaviour and late hospital presentation reflecting the operational realities of response to access to HIV services and retention in care in Sierra Leone. Such obstacles may be addressed by using patient-centred, individualized care techniques that bring HIV services closer to the PLHIV. Promoting PLHIV’s early health-seeking behaviour may also reduce the rate of disease progression. It contextualizes warnings from the International AIDS Society that West Africa lags behind in meeting the 95–95–95 global target [21, 22]. Therefore, the national HIV programs should redouble their efforts to address known barriers to access, such as the low HIV testing rate, low rates of HIV viral suppression, and improve retention rate to address disease progression, opportunistic infections, and comorbid conditions [23–28].

In our study, we present findings of AHD among young PLHIV aged 15–24 years who reported for routine HIV care at the outpatient HIV clinic and or self-referred for inpatient care Multiple predictive factors of AHD were noted among the entire study population and inpatients highlighting issues with access to care and significant challenges with retention in care among PLHIV aged 15–24 years. The high burden of AHD in both groups also supports the fact that CD4 + count and screening for opportunistic infections such as TB are recommended in PLHIV regardless of the setting in which they received care [29]. However, the declining trend in CD4 + count testing has been observed across sub-Saharan Africa, posing a risk of overlooking AHD diagnoses and potentially jeopardizing the effectiveness of antiretroviral therapy (ART) programs [27].

We found that age, adolescents aged 15–19 years was an independent predictor of AHD. Multiple studies [30–32] have highlighted the effect of age on AHD and emphasize disease progression not only in the elderly but particularly in newly diagnosed young people. This fact highlights the need for targeted HIV screening among young people and early treatment initiation to prevent HIV disease progression. Furthermore, an increased level of education was a protective factor against AHD; however, most of those aged 15–19 years could not have reached tertiary education, and therefore, age is a confounder. An increased level of education is a protective factor attributed to seeking medical services and a better understanding of instructions, including medication intervals and clinical follow-up visits, as have been reported in multiple studies [33–35]. Our study is also consistent with findings from other studies that report females to be less likely than men to present with AHD [36, 37].

TB is the most prevalent opportunistic infection among admitted YLHIV with AHD in this study, indicating the high incidence of TB in Sierra Leone, one of the 30 high TB burden nations in the world. [38, 39]. Hepatitis B co-infection rate of 6% reported in this study is synonymous with the national HBV endemicity of 8–10% and shares common transmission routes with that of HIV. This picture is a major concern and calls for a scale-up of health services in hepatitis B prevention programs to prevent immune reconstitution disease and hepatocellular carcinoma [39–41]. Furthermore, a recent hospital-based study [23] in Sierra Leone presented the pattern of stroke in HIV patients with lower CD4 cell counts. These findings highlight the need for improved co-infection screening and treatment among PLHIV, indicating that co-infections are important manifestations of AHD. Hence, there is a need for integrated, comprehensive care of comorbidities in HIV service delivery points in Sierra Leone.

Our study has strengths and limitations. First, this study provides data from a major tertiary hospital in Sierra Leone. Therefore, the findings cannot be generalised to all PLHIV aged 15–24 years in Sierra Leone. Second, given that the study was conducted at a referral facility, the tendency of referral bias cannot be excluded. More patients could have been referred to the hospital to seek care for AHD and associated symptoms, indicating that the reported prevalence could be higher in the group that reported to this facility than the general population. Furthermore, the study is limited by the unavailability of several routine laboratory investigations, including viral load, to monitor patient progress and functional assessment.

Nonetheless, this is the first study in Sierra Leone to explore the burden of AHD and its predictors in young PLHIV aged 15–24 years, calling for a more targeted approach to addressing gaps in health service delivery for YLHIV in Sierra Leone.

## Conclusion

We report a high prevalence of AHD among YLHIV in the national referral hospital in Sierra Leone. These findings highlight the need to strengthen public health measures and policies to address socioeconomic barriers limiting access to healthcare services, particularly for YLHIV. Through concerted efforts and multisectoral interventions for treatment optimization, we call for implementing measures to improve access to timely diagnosis and retention in care among YLHIV.

## Data Availability

Data access for academic use on request to the HIV Clinic at Connaught Hospital, where data will be made available subject to academic review and acceptance of a data-sharing agreement. Please contact lakoh2009@gmail.com or the corresponding author, mbaldeh@gmail.com. The anonymised dataset is available upon request; see the data availability section.

## Abbreviations

AHD: Advanced HIV disease
ART: Antiretroviral therapy
CD4 + Cell Count: Cluster of differentiation 4
HIV: Human immunodeficiency virus
PLHIV: People Living with HIV
SSA: Sub-Saharan Africa
TB: Tuberculosis
YLHIV: Youths (15–24 years) Living with HIV
